# A leakage-controlled and SHAP driven machine learning framework for paediatric respiratory disease classification using Indian hospital EHR data

**DOI:** 10.1186/s12911-026-03493-2

**Published:** 2026-04-17

**Authors:** Anusha Prashanth Shetty, Surendra Shetty, Pavan Hegde, Shwetha Shetty, Nagaraja Shetty

**Affiliations:** 1 Department of Master of Computer Applications, Nitte (Deemed to be University), NMAM Institute of Technology (NMAMIT), Nitte, Udupi, Karnataka 574110 India; 2 Department of Paediatrics, Father Muller Medical College and Hospital, Kankanady, Mangalore, Karnataka 575002 India; 3 Manipal Institute of Technology, Manipal Academy of Higher Education, Manipal, India

**Keywords:** Explainable AI, Paediatric diagnostics, Clinical machine learning, SHAP analysis, Electronic health records, TF-IDF, XGBoost, Class imbalance, SMOTE, Data leakage prevention, Feature selection, Clinical text analysis, SDG 3, SDG 9

## Abstract

**Background:**

Classification of paediatric respiratory diseases is important for timely diagnosis and decision-making. Real-world clinical data is often compounded by challenges of data leaking, class imbalance, and high dimensional data modalities, which might compromise the reliability, methodological rigour, and interpretability of the model.

**Methods:**

This research work introduces a leakage-controlled and explainable machine learning model for multiclass classification of respiratory diseases in children namely Bronchitis, Bronchopneumonia, Upper Respiratory Tract Infection (URTI), and Wheeze using both structured numerical and unstructured free-text information obtained from practical Electronic Health Records (EHRs). The dataset includes 1,121 paediatric cases sourced from an Indian hospital. A multi-step pre-processing pipeline was applied, including data quality filtering prior to partitioning train-only imputation, and a two-stage data leakage prevention mechanism. Numerical variables were standardized, and clinical free-text narratives were vectorized using Term Frequency–Inverse Document Frequency (TF–IDF) encoding. Class imbalance was handled using the Synthetic Minority Oversampling Technique (SMOTE) applied strictly within cross-validation folds to avoid contamination. SHapley Additive exPlanations (SHAP) were used on a Random Forest baseline to audit for diagnostic leakage and guide feature selection. The top 100 SHAP-ranked features were then used to train Logistic Regression, Random Forest, XGBoost and Stacking Ensemble models. SHAP ranking was performed exclusively on the training partition following train-test splitting, ensuring that feature selection was not influenced by test set information.

**Results:**

SHAP analysis confirmed that predictions were driven by clinically relevant features such as age, breathing difficulty days, Peripheral Oxygen Saturation (SpO₂), respiratory rate (RR), and serum bicarbonate. “Respiratory System” feature was identified as leakage-prone and was excluded from final model training. Random Forest achieved the highest hold-out test accuracy of 0.8578 (95% Confidence Interval (CI): 0.8089–0.9022), with XGBoost achieving the highest micro-averaged Area Under the Receiver Operating Characteristic Curve (AUROC) of 0.9706 and Area Under the Precision-Recall Curve (AUPRC) of 0.9285, computed using a one-vs-rest strategy. The narrow Cross Validation (CV) test gap is consistent with limited overfitting within this single-centre internal validation setting.

**Conclusion:**

The proposed pipeline supports transparent and leakage-controlled validation within a single-centre internal setting, contributing to methodological rigour in paediatric EHR-based Machine Learning (ML research). External validation across diverse clinical settings would be required before consideration of clinical deployment. The study also contributes to global health goals by supporting the development of equitable and reliable diagnostic technologies aligned with Sustainable Development Goals, particularly SDG 3 (Good Health and Well-being) and SDG 9 (Industry, Innovation, and Infrastructure).

**Supplementary Information:**

The online version contains supplementary material available at 10.1186/s12911-026-03493-2.

## Background

Paediatric respiratory diseases are a major health concern worldwide, responsible for childhood morbidity and mortality in India and across the globe [1, 2]. Entities such as wheeze/bronchiolitis, pneumonia, bronchitis, and Upper Respiratory Tract Infection (URTI) are becoming the leading causes of hospital visits [3, 4]. These require early diagnosis and appropriate treatment to enable timely intervention and prevent complications. However, these diseases often have overlapping symptoms, such as fever, cough and respiratory distress [3, 5], which often leads to misclassification and results in antibiotic misuse and delays in treatment [6]. It is also important to note the lack of availability of advanced imaging facilities in primary health care centres [7, 8]. Situation worsens in the case of paediatrics due to lack of their inability to express their symptoms [3].

Considering the limited availability of modern imaging facilities in many primary healthcare facilities, the information available from the Electronic Health Records (EHRs) plays a pivotal part in healthcare decision-making in paediatric patients. The EHRs include patient information such as demographics, vital statistics, lab results, and physician-generated free-text clinical narratives [9]. Machine learning (ML) approaches can be used for the systematic analysis of patient data, which might prove helpful in respiratory condition classification with the use of inherent patterns available in patient healthcare records.

Many studies have used EHR data for building classification models for paediatric respiratory diseases; most of the studies focused on using lung sounds and imaging techniques; purely text and numeric based research was comparatively less explored [10–13].

Though EHRs provide a rich source of multimodal information, they also introduce several methodological difficulties among the methodological challenges facing EHR-based clinical ML, diagnostic information leakage represents a particularly critical failure mode. Unlike high dimensionality or class imbalance which affect model efficiency and minority class performance leakage directly invalidates reported performance metrics by allowing label-related information to inadvertently enter the feature space. In text-rich EHR data, leakage pathways are especially difficult to control: diagnostic terminology may appear within free-text clinical narratives recorded before diagnosis, post-diagnostic documentation may be inadvertently included in training features, and preprocessing steps such as Term Frequency Inverse Document Frequency (TF-IDF) vocabulary construction or feature scaling may be fitted on data that includes validation or test partitions. A model trained under these conditions may appear highly accurate while learning to recognise diagnostic labels rather than underlying clinical patterns, rendering reported metrics unreliable despite strong benchmark performance [14–16].

Prior studies have made important individual contributions toward addressing these challenges. Lee et al. controlled diagnostic leakage through temporal splitting, using only pre-diagnosis clinical features, and reported Area Under the Receiver Operating Characteristic Curve (AUROC) of approximately 0.953 for paediatric lower respiratory tract infection classification [17]. Serin et al. utilised SHapley Additive exPlanations (SHAP) for feature selection prior to model training, achieving classification accuracies of 77–88% for paediatric pneumonia severity prediction [18]. Ma et al. applied **L**east **A**bsolute **S**hrinkage and **S**election **O**perator (LASSO) for feature selection with XGBoost achieving AUROC of approximately 0.98, with SHAP identifying key clinical predictors [19]. These works address individual pipeline components. However, to our knowledge based on our review of published literature the simultaneous integration of diagnostic keyword masking to prevent text-based leakage, per-fold preprocessor refitting to eliminate intra-cross-validation leakage, fold-wise synthetic sample control, and SHAP-based post-hoc leakage auditing within a unified framework has not been the primary focus of prior work in paediatric respiratory EHR classification, to our knowledge based on our review of published literature this represents the first application of such a unified leakage-controlled framework to an Indian paediatric respiratory EHR dataset.

Our study presents a leakage-controlled multimodal machine learning framework for four-class paediatric respiratory disease classification using real-world EHR data collected from a single tertiary care centre in India. The framework makes the following specific contributions:

1. Proactive diagnostic keyword masking combined with reactive SHAP-based leakage auditing to prevent and detect text-based label leakage.
2. Per-fold preprocessor refitting within cross-validation ensuring TF-IDF vocabulary construction, feature scaling, and imputation are performed exclusively on training partitions.
3. Within-fold SMOTE oversampling preventing synthetic data contamination of validation partitions.
4. SHAP-based dimensionality reduction from a high-dimensional mixed-modal feature space.
5. Multimodal integration of structured clinical measurements with unstructured free-text narratives for paediatric respiratory disease classification using an Indian hospital EHR dataset

## Methods

### Study design and data collection

In our study, we analysed a novel real-world paediatric dataset comprising of EHRs collected retrospectively using continuous case sampling approach from Father Muller Medical College and Hospital between 2015 and 2021, after obtaining approval from the Institutional Review Board (Protocol No: 110/2020 (FMIEC/CCM/279/2024)). In all, 1,121 paediatric cases were included, each corresponding to a unique patient admission verified using patient identifiers no child contributed more than one record to the dataset. Clinical diagnoses had been assigned at the point of care by qualified clinicians, and cases were selected during data extraction using predefined International Classification of Diseases (ICD) code groupings rather than reclassified post hoc. Cases were distributed across four diagnostic categories wheeze (47.3%), upper respiratory tract infection(URTI) (24.0%), bronchopneumonia (24.0%), and bronchitis. (4.7%). All records were anonymised and preprocessed following institutional ethical guidelines. The dataset initially consisted of 63 variable per record, ranging from demographic, anthropometric, clinical, laboratory, and narrative text categories. A step of privacy-compliant filtering excluded personally identifiable information, such as registration numbers, timestamps, and geographic identifiers. and treatment-related fields, for example, discharge summaries, radiology references that might indirectly reveal diagnostic results. Upon filtering, 58 usable variables were retained across numeric and free-text domains for further preprocessing, which are listed in (Table 1). 44 variables were finalized after preprocessing listed in (Table 2) the Respiratory System (RS) variable was dropped due to label leakage making the final variable count 43. Following TF-IDF vectorization of the free-text variables, these 43 variables expanded into approximately 1,092 individual features in the model-ready feature space. From this expanded feature space, the top 100 features ranked by SHAP importance were selected for final model training. These 100 features represent a subset of the vectorized feature space, not a subset of the original 43 variables a distinction important for interpreting the SHAP analysis results. SHAP ranking was performed on the training partition only, following the 80–20 train-test split, and the resulting feature subset was applied consistently across all subsequent cross-validation folds without re-ranking. 

### Data preprocessing framework

**Fig. 1:**
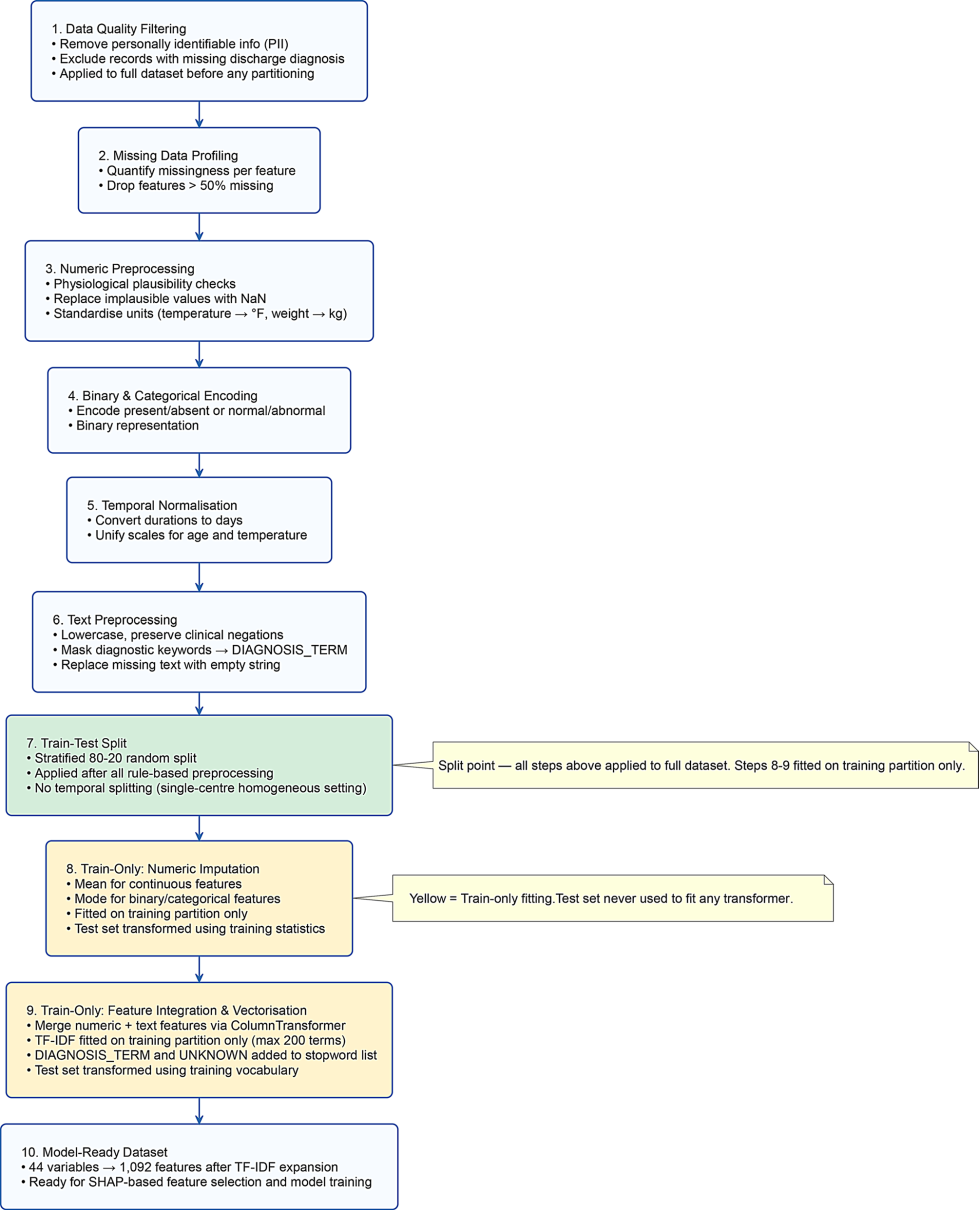
Overview of the data preprocessing pipeline including ethical filtering, imputation, normalization, and feature integration

A robust multi-stage preprocessing framework (see Fig. 1) was implemented to ensure data consistency, eliminate label leakage, and maintain the semantic integrity of clinical information.

**Table 1:** List of clinical and laboratory variables used for preprocessing

Category	Variables
Demographic	Present Age, Sex
Clinical Symptoms	Fever days, Cough/Cold days, Breathing difficulty days, history of presenting illness
Birth and Development	Natal, Birth Weight (kg), Postnatal/Neonatal Intensive Care (NICU), Developmental History, Immunization History
Physical Measurement	Length(cm), Weight(kg), Child Active / Alert
General Examination	Pallor, Icterus, Cyanosis, Clubbing, Lymphadenopathy, Oedema
Vital Signs	Pulse Rate (Beats per minute) (PR (bpm)), Temperature (Temp(F)), Systolic Blood Pressure (BP), Diastolic BP, Respiratory Rate (RR) (cycles/min), Peripheral Oxygen Saturation (SpO2) (% at room air), Capillary Filling Time (CFT)
Ear, Nose and Throat (ENT) Examination	Ears, Nose, Throat
Systemic Examination	Respiratory System (RS), Cardiovascular System (CVS), Per Abdomen (P/A), Central Nervous System (CNS)
Hematology	Haemoglobin (g/dl), Leukocyte Count Total (/cumm), Platelet Count (/cumm), Neutrophils (%), Lymphocytes (%), Eosinophils (%), Monocytes (%), Basophils (%)
Biochemistry	Serum Creatinine (mg/dl), Serum Sodium (mEq/L), Serum Potassium (mEq/L), Serum Chloride (mEq/L), Serum Bicarbonate (mEq/L), Serum C reactive Protein (CRP (mg/l)), Glucose
Arterial Blood Gas	Potential of Hydrogen (pH), partial Pressure of Carbon Dioxide (PCO_2_ (mmHg)), Partial Pressure of Oxygen (PO_2_(mmHg)), Bicarbonate (HCO_3_−) (mmol/L), Total Carbon Dioxide (TCO_2_ (mmol/L)), Sulphur Dioxide (SO_2_ (%)), Lactate

### Missing data profiling

Feature-wise missingness was quantified as shown in Eq. (1)

1 $$Missing\% = \frac{{MissingCount}}{{TotalCount}} \times 100$$

Following standard data-preprocessing guidelines for EHR-based datasets [20], features exceeding 50% missingness were excluded.

To ensure that physiological measurements from different patients were comparable and that implausible values did not distort model learning, the following standardisation steps were applied.

### Numeric standardisation and outlier treatment

Continuous variables, including vital signs, anthropometric data, and serum biochemistry, were standardised after physiological plausibility checks. Implausible readings beyond the 0.1–99.9 percentile bounds or outside known paediatric reference ranges were replaced with NaN and subsequently imputed. Unit discrepancies were harmonised (e.g., temperature→ Fahrenheit, weight → kilograms).

### Binary and categorical encoding

Binary clinical indicators such as pallor, cyanosis, or oedema were encoded as depicted in Eq. (2)

2 $${X_{binary{\text{ }} = }}\left\{ {\begin{array}{*{20}{c}} {1,\,if\,abnormal\,or\,present} \\ {0,\,if\,normal\,or\,absent.} \end{array}} \right.$$

### Temporal and unit normalisation

Duration-related features (e.g., fever days, cough duration) were standardised into days through unit conversion (weeks×7, months×30). All numeric temperature and age measurements were normalised to consistent scales for cross-patient comparability.

### Text preprocessing

Free-text narratives (chief complaints, developmental history, system examinations) were minimally pre-processed to preserve medical negations and contextual cues. Steps included lowercasing, whitespace normalisation, and removal of encoding artefacts; while excluding stemming or lemmatisation to retain interpretive clarity and the missing fields were replaced by placeholders UNKNOWN which was eventually added TF-IDF stop word list ensuring it does not appear in the final feature list.

After completing the above preprocessing steps, the dataset was finalised with 33 numeric and 11 textual clinical variables as listed in Table 2.

**Table 2:** Text and numeric features after completion of the first stage of preprocessing

Numeric Features	Text Features
Age Months	Sex
Pallor	Developmental History
Cyanosis	Immunization History
Clubbing	Ears
Odema	Nose
Lymphadenopathy	Throat
Icterus	RS
PR (bpm)	CVS
RR (cycles/min)	P/A
SpO2%	CNS
Weight (kg)	Clinical History
Height(cm)	
Haemoglobin (g/dL)	
Leukocyte Count (cumm)	
Platelet Count	
Neutrophils Percent	
Lymphocytes Percent	
Eosinophils Percent	
Monocytes Percent	
Basophils Percent	
Breathing difficulty days	
Cough/Cold Days	
Fever days	
Temperature(F)	
birth weight (kg)	
Serum Creatinine (mg/dl)	
Serum Sodium (mEq/L)	
Serum Potassium (mEq/L)	
Serum Chloride (mEq/L)	
Serum Bicarbonate (mEq/L)	
Serum Crp (mg/l)	

### Data partitioning and leakage control

To mitigate the risk of diagnostic information leakage, a two-stage leakage control framework Proactive Masking and Reactive Masking was implemented before model training.

### Proactive masking

Diagnostic keywords directly or indirectly describing the target labels, such as wheeze, bronchitis, bronchopneumonia, URTI, and their morphological variants, were programmatically replaced with a neutral placeholder token.

(DIAGNOSIS_TERM). The complete masking lexicon including all masked terms, morphological variants, and abbreviations is provided in Supplementary Material (Table S1). This ensured that the text-based model learned from the clinical context rather than explicit label references. Eventually the DIAGNOSIS_TERM was added to TF-IDF stop word list to ensure it does not appear in the final feature list.

### Reactive filtering

The Respiratory System examination (RS)text feature was found to have a significant impact on model choices during SHAP-based interpretability analysis. Inspection revealed that it contained direct diagnostic phrases that were indirectly leaking the diagnostic label, such as wheeze present and bronchial breath sounds. All subsequent model training and evaluation did not include the RS column in order to avoid this type of implicit label encoding [21, 22].

This combined proactive-reactive approach follows clinical machine learning best practices by ensuring that features represent pre-diagnostic evidence within this single-centre internal validation setting [23–25]. Both leakage control steps proactive masking and reactive exclusion of the RS variable were applied before data partitioning. Proactive masking was applied uniformly to the full dataset prior to any train-test split, as it is a rule-based lexicon substitution that involves no learning from data and introduces no statistical dependence between partitions. Reactive exclusion of the RS variable was determined through SHAP analysis on training data only. Numerical imputation and feature scaling were fitted exclusively on the training partition and applied to the test set using training-derived statistics only. No filtering, resampling, or data-driven transformation was applied to the test set independently.

### Feature vectorization and integration

Following preprocessing, the structured and unstructured attributes were transformed into a unified feature space suitable for machine learning using a hybrid Column Transformer architecture. To enable the model to learn simultaneously from structured clinical measurements and unstructured physician narratives.

Numeric and textual attributes were processed independently:

Continuous and categorical numeric features were retained in their standardised form. The missing features were imputed using standard statistical imputation techniques mean substitution for continuous features and mode for categorical features (see Eq**.** (3)), a widely accepted approach in clinical and EHR-based machine-learning studies [26, 27]. The imputation statistics were derived exclusively from each fold’s training partition and never from validation or test data.

3 $${X_{i,j = }}\left\{ {\begin{array}{*{20}{c}} {mean({x_j}\,|y\, = {y_i}),\,for\,continuous\,features} \\ {mode({x_j}\,|y\, = \,{y_i}),\,for\,categorical\,features.} \end{array}} \right.$$

Each free-text column (e.g., clinical history, system examinations) was vectorized using the Term Frequency–Inverse Document Frequency (TF–IDF) method, to a maximum of 200 terms per column to minimise sparsity.

The DIAGNOSIS_TERM masking placeholder and the UNKNOWN missing-value token were explicitly added to the TF–IDF stopword list, ensuring neither token appeared in the final feature space.

The TF–IDF transformation assigned each term a weight using Eq. (4).

4 $${w_{i,j}} = t{f_{i,j}} \times log\left( {\frac{N}{{d{f_j}}}} \right)$$

Where w_i, j_ denotes the TF–IDF weight of term j in document i, tf_i, j_ represents its term frequency, and df_j_ is the document frequency. This process yielded approximately 1,187 features across 44 columns, forming the complete model-ready dataset.

### Class imbalance handling

In order to address data imbalance, and to prevent the model from being dominated by majority classes at the expense of clinically important minority conditions especially, underrepresentation of bronchitis (4.7%), the standard SMOTE with k nearest neighbours set to the default value of 5 was applied [28] after SHAP-based feature selection, operating on the reduced 100-feature space within each fold’s training partition, SHAP ranking was performed exclusively on the training partition following train-test splitting, ensuring that feature selection was not influenced by test set information at any stage. Standard SMOTE was selected after considering its usage as standard baseline in clinical tabular data [29–31], and because tree-based ensemble methods (RF, XGBoost) have frequently been used with SMOTE in medical datasets and often benefit from it [31–34]. SMOTE was applied within each fold of cross-validation, rather than across the full dataset. The fold-wise application was done to ensure that synthetic samples were confined to training subsets, thus preventing any data leakage into validation or test partitions and maintaining the integrity of performance estimates.

### SHAP-based leakage detection and model interpretability

Beyond feature importance ranking, SHAP analysis served a clinical validation function verifying that model decisions reflected pre-diagnostic clinical evidence rather than incidentally encoded diagnostic labels.

SHAP analysis was used to ensure transparency and detect possible sources of diagnostic information leakage using Random Forest as base classifier; because of its resilience to noise, capacity to capture nonlinear feature interactions, and compatibility with the TreeExplainer algorithm, which effectively calculates ensemble tree Shapley values [35–37]. The mean absolute SHAP value for each feature j across all n training samples was used to quantify its contribution to model predictions using Eq. 5:

5 $$\:{I}_{j}=\frac{1}{n}\sum\:_{i=1}^{n}|{\phi\:}_{i,j}$$

Here $\:{\phi\:}_{i,j}$ Represents the SHAP contribution of feature j for instance i.

The key objectives of SHAP analysis were as follows:

### Leakage detection

SHAP value inspection revealed that the RS feature had unusually high importance scores simply because of the presence of explicit diagnostic terms such as “wheeze present” and “crepitus heard” This indicated information leakage and thus was excluded from all further proceedings.

### Model evaluation and interpretability

SHAP summaries highlighted clinically meaningful predictors such as age, Peripheral Oxygen Saturation (SpO2), and Respiratory Rate, in accordance with established diagnostic reasoning in paediatric respiratory diseases [38–40].

The correspondence between the SHAP-derived most important features and known clinical predictors offers further evidence that the model’s predictions were primarily driven by authentic clinical patterns rather than documentation artifacts. SHAP was employed as a post-hoc analysis tool to assess feature contributions and identify potentially suspicious correlations. However, SHAP results cannot be taken as definitive evidence to preclude the role of proxy variables, nor can they validate causality or rule out the presence of all possible leakage paths, and therefore these results should be considered as supporting evidence for model interpretability rather than definitive proof of the absence of leakage.

### Feature refinement using SHAP rankings

In addition to leakage detection and interpretability, SHAP values were used to guide feature refinement. The mean absolute SHAP importance scores were computed from a baseline Random Forest model trained on the full 1,092-feature space following the 80–20 stratified train-test split and prior to any final model training. Feature ranking was therefore performed entirely within the training data; the resulting top 100 SHAP-ranked features were then used consistently across all cross-validation folds, with all preprocessing transformations (TF-IDF fitting, imputation, and scaling) refitted independently within each fold's training subset. The top 100 SHAP-ranked features were selected to form the reduced feature set, **The complete list of selected features with corresponding SHAP importance scores is provided in Supplementary Material (Table ****S2****)**. following prior clinical machine learning studies that employ SHAP-based ranking to identify a limited subset of influential features for efficient modelling [41–45]. In our dataset, this threshold captured 76.0% of cumulative SHAP importance, with the importance curve flattening substantially beyond this point (**Supplementary Material (Fig. ****S1**)). SMOTE was subsequently applied to this reduced feature space within each cross-validation fold before model training. This ensured that the classifiers were trained on a compact, high-signal, leakage-controlled subset of multimodal EHR features, reducing the risk of artefactual feature influence within this internal validation setting.

### Model training and evaluation framework

All modelling decisions were designed around two clinical priorities: ensuring that performance estimates reflect genuine generalisation rather than preprocessing artefacts, and maintaining minority class representation throughout validation.

Four Models, namely Logistic Regression, Random Forest, XGBoost, and Stacking Ensemble, were trained using a stratified and leakage-controlled experimental design after preprocessing and SHAP-based feature refinement. A stratified 80–20 train–test split was applied on the dataset. Patients were randomly allocated to training (80%) and hold-out test (20%) partitions using stratified sampling to preserve class proportions across both sets. No temporal splitting was applied, as the dataset originates from a single paediatric centre with a homogeneous clinical workflow throughout the 2015–2021 collection period, and no systematic shifts in diagnostic practice or documentation were identified across this interval. All pre-processing steps, including TF-IDF fitting and SMOTE over-sampling, were performed strictly within the training folds to avoid information leakage. All the models were trained and validated using a 5-fold Stratified K-Fold Cross-Validation, ensuring reproducibility and consistent representation of minority classes across folds.

Full library versions, hyperparameter settings, are provided in Supplementary Methods.

### Performance evaluation metrics

The Model performances were evaluated using both global and class-specific metrics to ensure diagnostic reliability across all disease categories. Some of the key metrics are as follows:

- Accuracy – provides the overall proportion of correctly classified samples.
- Precision, Recall, and F1-Score – evaluates per class and macro-average, which accounts for data imbalance.
- Confusion Matrix – helps to visualise misclassifications.
- Cross-Validation vs. Test Gap – difference between mean CV accuracy and hold-out test accuracy, useful to assess generalisation stability.

## Results

### Model performance overview

Model performance was evaluated using Five-fold stratified cross-validation and independent testing on the 20% hold-out set. The results across all 4 models are listed in Table 3, which provides a summary of the model performance.

including bootstrap 95% confidence intervals (CI) computed from 1,000 resamples of the hold-out test set to quantify uncertainty around point accuracy estimates.

Random Forest achieved the highest hold-out test accuracy of 0.8578 (95% CI: 0.8089–0.9022), followed by the Stacking Ensemble at 0.8489 (95% CI: 0.7999–0.8933), XGBoost at 0.8444 (95% CI: 0.7956–0.8889), and Logistic Regression at 0.7911 (95% CI: 0.7377–0.8400). Cross-validation accuracies were consistent with hold-out test performance, with CV–test gaps ranging from 0.0065 to 0.0172, suggesting limited overfitting within this internal validation setting. These gaps alone do not demonstrate generalisation beyond this cohort; external or temporal validation would be required to substantiate broader robustness claims.

AUROC and Area Under the Precision–Recall Curve (AUPRC) were computed using a one-vs-rest strategy with both micro-averaged and macro-averaged values reported. XGBoost achieved the highest micro-averaged AUROC of 0.9706 and AUPRC of 0.9285, followed by the Stacking Ensemble (AUROC: 0.9688, AUPRC: 0.9261), Random Forest (AUROC: 0.9613, AUPRC: 0.9155), and Logistic Regression (AUROC: 0.9487, AUPRC: 0.8749). The high discrimination metrics likely reflect the relatively distinct clinical presentations of the four diagnostic categories within this single-centre paediatric cohort and should be interpreted cautiously in the absence of external validation.

To verify that model performance was not driven by the DIAGNOSIS_TERM masking token or TF-IDF text artefacts, a structured-only sensitivity analysis was conducted training models on numeric features only without text. Test accuracies were 0.7600 (Logistic Regression), 0.8222 (Random Forest), and 0.8311 (XGBoost), compared to 0.7911, 0.8578, and 0.8444 respectively for the full multimodal models. The consistent positive deltas (range: +0.013 to + 0.036) confirm that text features contribute a modest but consistent improvement, and that performance is primarily driven by structured clinical variables.”

**Table 3:** Model performance result

Model	CV-Accuracy	Test Accuracy	95% CI	CV-Test Gap
Logistic Regression	0.7846 ± 0.0396	0.7911	[0.7377–0.8400]	0.0065
Random Forest	0.8661 ± 0.0152	0.8578	[0.8089–0.9022]	0.0083
XGBoost	0.8616 ± 0.0227	0.8444	[0.7956–0.8889]	0.0172
Stacking Ensemble	0.8650 ± 0.0174	0.8489	[0.7999–0.8933]	0.0161

### Class-wise evaluation

**Fig. 2:**
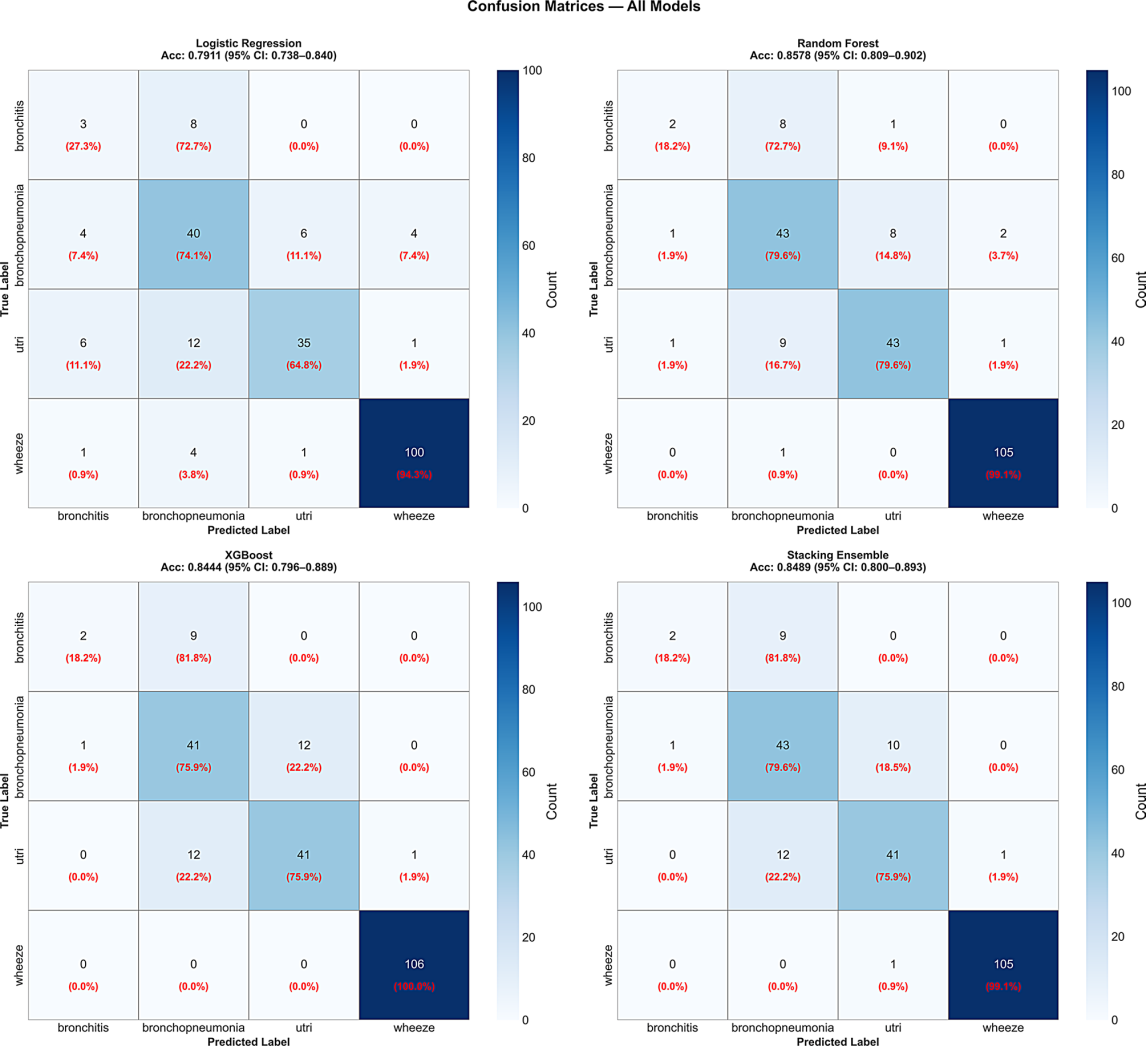
Confusion matrix showing classification distribution across four respiratory disease categories for all four models

All the models performed consistently well across all four respiratory disease categories, with notable variation by class reflecting the underlying data distribution. Detailed class-wise metrics are presented in Table 4 and the confusion matrices are illustrated in Fig. 2.

Wheeze, the majority class (47.3%), achieved the highest recall across all models, ranging from 0.94 to 1.00, consistent with its strong representation in the training data. Bronchopneumonia and URTI both achieved high recall across models, reflecting their balanced representation and clinically distinct feature profiles. Bronchitis, the minority class (*n* = 53, 4.7%), showed consistently lower recall ranging from 0.18 to 0.27 across all models despite fold-wise SMOTE application. This represents a meaningful limitation of the current framework the absolute size of the bronchitis class constrains achievable recall regardless of resampling strategy. Future work should explore cost-sensitive learning or targeted augmentation approaches specifically calibrated for this class.

Based on the confusion matrix results, it was revealed that **(**Fig. 2), most misclassifications occurred between bronchitis and URTI. This overlap reflected clinical reality, where these two conditions often share similar early symptoms such as brief fever and mild cough, making them challenging to distinguish even for physicians [46, 47]. This finding suggests that additional discriminative features specific to bronchitis, such as auscultatory findings or duration of symptoms, may need greater weight in future model iterations.

In addition to the overlap between bronchitis and URTI, wheeze had near-perfect classification accuracy across all models, as expected given its majority class status and clear presentation including characteristic expiratory findings. Bronchopneumonia occasionally overlapped with URTI, likely due to similarities in early presentations prior to consolidation. No systematic pattern of bias towards any single diagnosis was detected within this internal validation setting; however, the consistently low recall for the bronchitis class reflects the constraints imposed by its limited sample size and should be interpreted as a meaningful limitation rather than evidence of balanced performance across all classes. Misclassifications were otherwise generally interpretable in a clinical context.

**Fig. 3:**
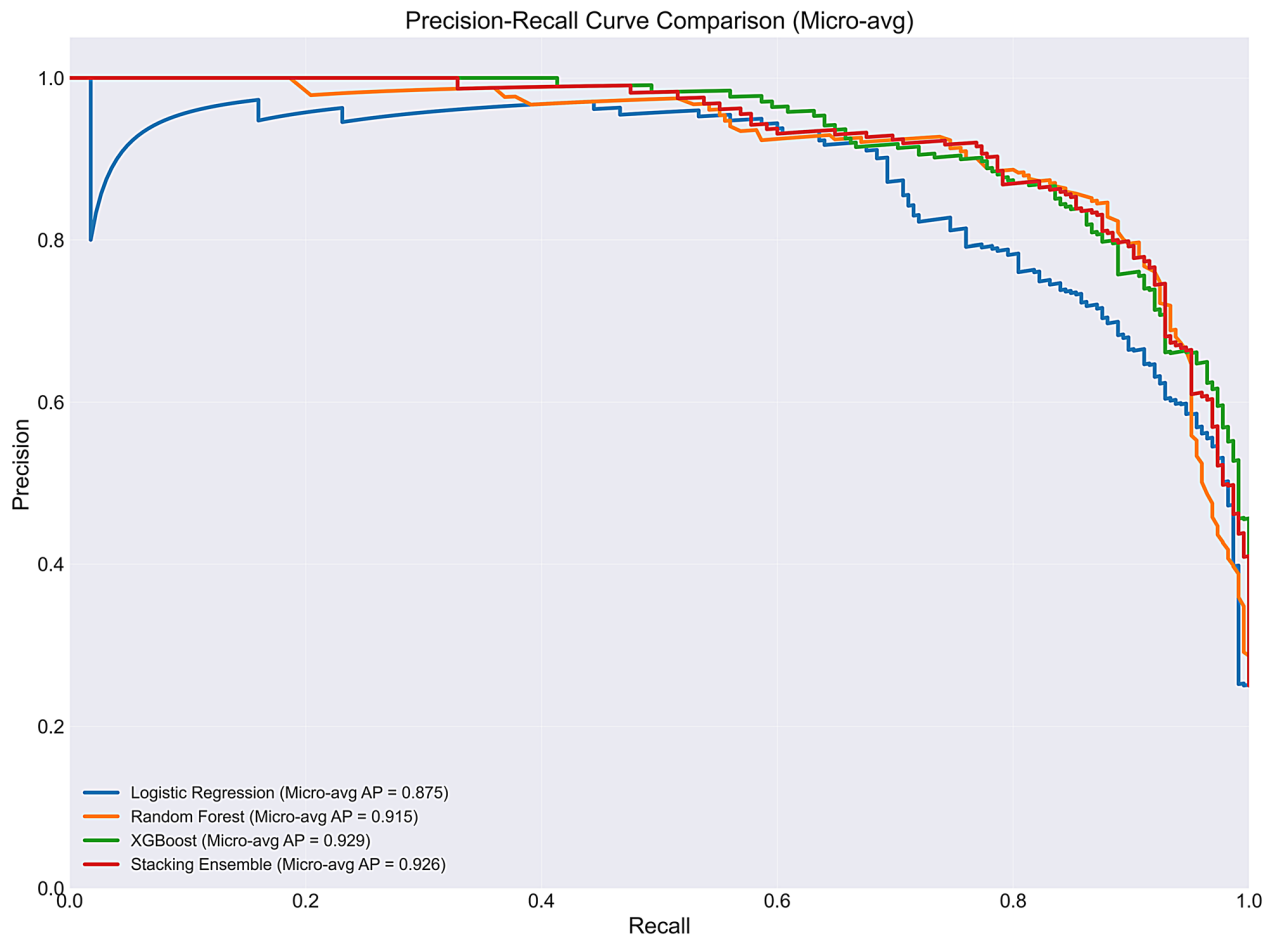
Precision–Recall (PR) curve comparison across all models

**Fig. 4:**
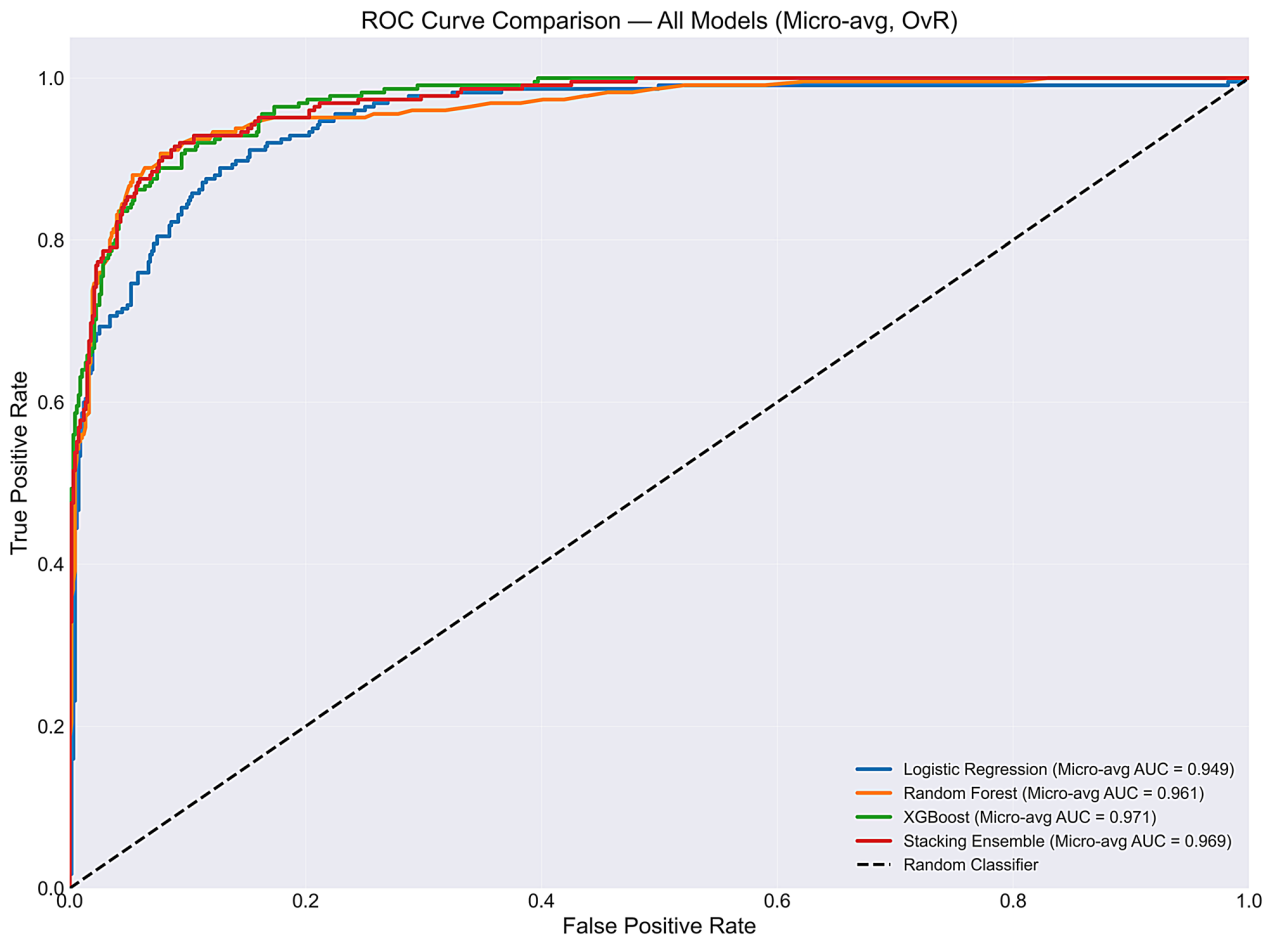
Comparison of Receiver Operating Characteristic (ROC) curves for all four classifiers

To assess overall discriminative ability, both Receiver Operating Characteristic Curve (ROC) and Precision–Recall curves were generated for all models (Fig. 3 and Fig. 4).

Class-wise AUROC computed using a one-vs-rest strategy is presented in Table 4. For the minority bronchitis class, One versus Rest (OvR) AUROC ranged from 0.867 (Stacking Ensemble) to 0.893 (Logistic Regression and Random Forest), indicating reasonable discriminative ability at the probability level despite low recall. Wheeze achieved near-perfect OvR AUROC across all models (0.986–0.998), while bronchopneumonia and URTI ranged from 0.894 to 0.949.

**Table 4:** Class-wise AUROC result

Class	Logistic Regression	Random Forest	XGBoost	Stacking Ensemble
Bronchitis (minority)	0.893	0.893	0.879	0.867
Bronchopneumonia	0.904	0.894	0.924	0.925
URTI	0.929	0.949	0.946	0.942
Wheeze	0.986	0.997	0.998	0.998
Micro-avg	0.949	0.961	0.971	0.969
Macro-avg	0.928	0.933	0.937	0.933

### SHAP-based model interpretability

**Fig. 5:**
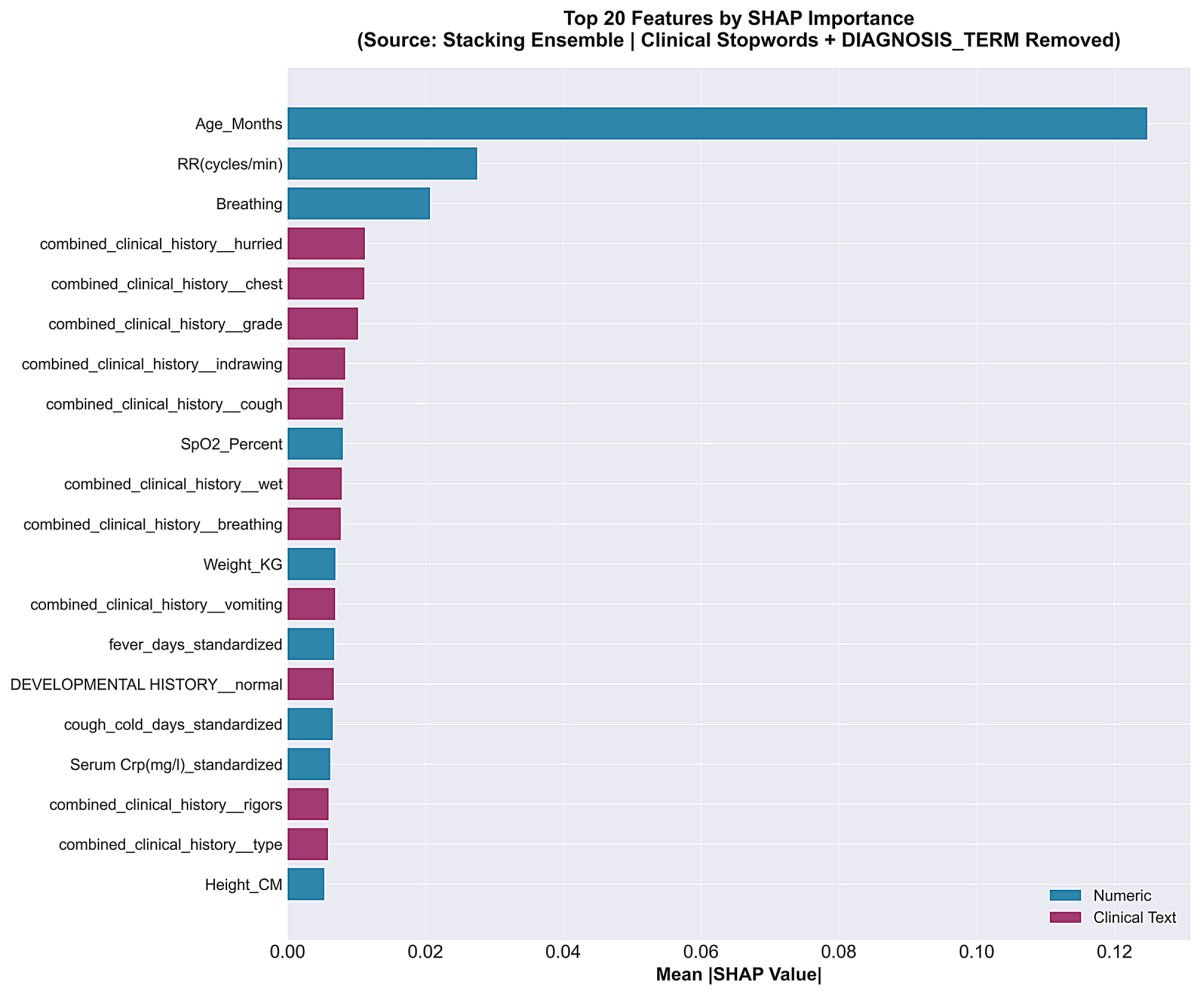
SHAP summary plot illustrating feature importance distribution

To confirm the significance of individual features and make sure that no diagnostic data was directly affecting the model performance, the SHAP analysis was employed using the Random Forest sub-estimator of the final Stacking model. Both text-based and numerical clinical variables contributed significantly to the prediction of respiratory diseases, as the SHAP summary plot (Fig. 5) illustrates. Some of the most significant predictors were Serum bicarbonate levels, age, respiratory rate, and oxygen SpO₂ all clinically plausible indicators of paediatric respiratory disease severity and consistent with established diagnostic reasoning in this population [38–40].

Furthermore, specific terms that were taken from the clinical notes helped reveal some of the contextual information supporting accurate model predictions. The alignment of top SHAP-ranked features with established clinical reasoning provides evidence that model decisions were primarily driven by legitimate clinical signals rather than documentation artefacts. However, SHAP explanations cannot conclusively rule out the influence of proxy variables, confirm causal validity, or guarantee the absence of all indirect leakage pathways. These explanations should therefore be interpreted as supporting evidence for model transparency rather than definitive proof of leakage absence.

## Discussion

Our work proposes a leakage-controlled multimodal machine learning framework for pediatric respiratory disease classification, leveraging real-world electronic health record data from a single tertiary care center. The proposed framework combines proactive diagnostic keyword masking, reactive SHAP-based leakage detection, fold-wise SMOTE oversampling, and SHAP-guided feature refinement into a unified cross-validation framework.

Previous studies on paediatric respiratory disease classification have majorly used deep learning or traditional ML models, often reporting high classification accuracy or AUROC values. For example, Yu et al. and Liang et al. applied NLP-based deep learning techniques to EHR data and achieved strong predictive performance [3, 48]. However, explicit strategies for leakage-controlled validation, synthetic sample auditing, or interpretability-driven verification were not the primary focus of these studies.

Ensemble based approaches have also been widely adopted due to their strong predictive capabilities. Lee et al. developed a Random Forest model using structured clinical data and reported AUROC values of about 0.953 [17].

Interpretability has become an essential requirement for clinical ML systems, Studies by Serin et al. [18]and Ma et al. [19] demonstrated the utility of SHAP analysis for feature attribution and explanation. Serin et al. [18] employed SHAP for feature reduction prior to training and achieved classification accuracies between 77% and 88% using ensemble models.

These works represent meaningful methodological contributions. The present study differs not by claiming these approaches are absent from the literature, but by integrating diagnostic keyword masking, fold-wise synthetic sample control, per-fold preprocessor refitting, and SHAP-based leakage auditing simultaneously within a unified cross-validation framework a combination that, to our knowledge based on our review of published literature, has not been the primary focus of prior work in paediatric respiratory EHR classification.

The SHAP analyses identified clinically meaningful variables such as age, oxygen saturation (SpO₂), duration of breathing difficulty, and serum bicarbonate as key contributors to model predictions. These findings are consistent with prior studies by Liu et al. [49] and Kumar et al. [50], which also reported the importance of physiological and clinical markers in paediatric respiratory disease modelling. The correlation between model explanations and established clinical knowledge enhances clinician confidence and supports the interpretability of the proposed framework. However, SHAP explanations cannot conclusively rule out the influence of proxy variables, confirm causal validity, or establish the absence of all indirect leakage pathways. These explanations should therefore be interpreted as supporting evidence for model transparency rather than definitive proof of leakage absence.

The observed test accuracies of 0.79–0.86 and micro-averaged AUROC of 0.949–0.971 should be interpreted within the context of single-centre internal validation. These metrics likely reflect the relatively distinct clinical presentations of the four diagnostic categories within this specific paediatric cohort and represent an optimistic upper bound on expected performance. A narrow CV–test gap alone does not demonstrate generalisation or robustness across settings external or temporal validation across diverse institutions and patient populations would be required to substantiate such claims. Bootstrap 95% confidence intervals are reported to quantify uncertainty around point estimates, though these do not substitute for external validation.

From a clinical perspective SHAP based explanations enables clinicians to inspect feature contributions at both population and individual patient levels, facilitating clinical sense making rather than opaque prediction. Such interpretability is increasingly recognised as a requirement for the safe integration of artificial intelligence (AI) into clinical workflows, particularly in paediatric settings where diagnostic accountability is critical [51, 52].

However, the framework presented here has not been prospectively evaluated in a clinical setting, and its practical utility for decision support remains to be established through prospective studies. No subgroup fairness analysis was performed and claims regarding fairness or equitable performance across demographic groups are not made in this study.

More broadly, this study contributes to ongoing discussions surrounding methodological rigor in clinical machine-learning research. Performance inflation caused by data leakage or improper handling of synthetic samples remains a persistent concern in EHR based studies. The combined approach of proactive leakage prevention and post-hoc interpretability-based auditing may offer a useful methodological reference for future clinical prediction studies, though its applicability across other clinical contexts requires further empirical evaluation.

Several limitations should be acknowledged. First, bronchitis - the minority class (4.7%) - showed consistently low recall of 18–27% across all models despite fold-wise SMOTE application. however, alternative resampling or cost-sensitive learning strategies may further enhance minority class performance in future work.

Second, this study is limited to single-centre internal validation. Generalisability to other populations, clinical settings, or documentation practices cannot be assumed without multi-centre or temporal external validation.

Third, a random rather than temporal split was employed given the homogeneous single-centre data collection setting; temporal validation remains an important future direction.

Fourth, free-text clinical narratives were represented using TF–IDF features, selected for their robustness and transparency. While effective for the present study, contextual language models such as ClinicalBERT or BioBERT may further improve semantic representation and generalisability across diverse clinical documentation styles.

Fifth the top 100 SHAP-ranked features captured 76.0% of cumulative importance, higher thresholds were not systematically evaluated in this study due to the unfavourable sample-to-feature ratio at larger feature counts (896 training samples), the computational cost of per-fold preprocessing within the stacking ensemble, and the modest performance delta observed between structured-only and multimodal models suggesting diminishing returns beyond the selected threshold. Evaluating alternative thresholds remains a direction for future work.

Finally, although SHAP-based auditing provides valuable interpretability and explicit leakage inspection, it introduces additional computational overhead. Lightweight models or SHAP approximation strategies may be explored for large-scale or real-time deployment. The use of data from a single tertiary care hospital enabled controlled validation and detailed bias analysis; however, multi-centre validation across diverse populations will be essential to assess generalisability and fairness. Future work integrating multimodal data sources, such as radiological imaging or physiological signals, may further strengthen the clinical usage of the proposed framework.

## Conclusion

Our study produced a ML framework for classifying paediatric respiratory diseases, in which both numerical and textual clinical data were utilised for model development. Using a dataset of paediatric cases from an Indian hospital, we followed strict preprocessing, feature selection, and validation procedures to address key challenges in clinical machine learning, including data leakage, class imbalance, and model interpretability. These measures ensured that model decisions were driven by clinically meaningful evidence rather than artefacts of data processing. SHAP analysis proved valuable not only for feature selection but also for explaining model behaviour. Among the models evaluated, Random Forest achieved the highest hold-out test accuracy of 0.8578 (95% CI: 0.8089–0.9022), with XGBoost achieving the highest micro-averaged AUROC of 0.9706. CV–test gaps ranged from 0.0065 to 0.0172, suggesting limited overfitting within this internal validation setting. This study shows that careful and clinically informed design choices can turn routine electronic health record data into useful diagnostic information. By providing supporting evidence that model predictions are primarily driven by legitimate clinical features rather than artefacts of data processing, the proposed framework supports more reliable performance estimation in EHR-based clinical ML research within this single-centre internal validation setting. Such approaches contribute to methodological rigour in clinical ML research. External validation across diverse clinical settings would be required before consideration of clinical deployment.

## Supplementary Information

Below is the link to the electronic supplementary material.

Supplementary Material 1

## Data Availability

The data used in this study were obtained from electronic health records collected at Father Muller Medical College and Hospital. Due to privacy, ethical, and legal restrictions related to potentially sensitive patient information, the data cannot be made publicly available. The model code supporting the findings of this study can be provided upon request.

## Abbreviations

AI: Artificial Intelligence
AUPRC: Area Under the Precision-Recall Curve
AUROC: Area Under the Receiver Operating Characteristic Curve
BP: Blood Pressure
CFT: Capillary Filling Time
CNS: Central Nervous System
CRP: C reactive Protein
CV: Cross Validation
CVS: Cardiovascular System
EHR: Electronic Health Records
ENT: Ear, Nose and Throat
ICD: International Classification of Diseases
LASSO: Least Absolute Shrinkage and Selection Operator
LRTI: Lower Respiratory Tract Infection
ML: Machine Learning
NICU: Neonatal Intensive Care
NLP: Natural language Processing
ovR: One versus Rest
pH: Potential of Hydrogen
P/A: Per Abdomen
PCO2 (mmHg): Partial Pressure of Carbon Dioxide
PO2 (mmHg): Partial Pressure of Oxygen
TCO2: Total Carbon Dioxide
PR (bpm): Pulse Rate ( Beats per minute)
ROC: Receiver Operating Characteristic Curve
RR: Respiratory Rate
RS: Respiratory System
SO2: Sulphur Dioxide
SpO_2_: Peripheral Oxygen Saturation
SHAP: SHapley Additive exPlanations
SMOTE: Simple Minority Over Sampling Technique
TCO2: Total Carbon Dioxide
Temp: Temperature
TF-IDF: Term Frequency Inverse Document Frequency
URTI: Upper Respiratory Tract Infection
