# Supplementary material for: A leakage-controlled and SHAP driven machine learning framework for paediatric respiratory disease classification using Indian hospital EHR data
Source: BMC Med Inform Decis Mak. 2026 Apr 17;26:198. doi: 10.1186/s12911-026-03493-2 (PMC13227820; doi:10.1186/s12911-026-03493-2)
Supplement: Supplementary file 1 — Supplementary Material 1 [file 12911_2026_3493_MOESM1_ESM.docx]

**Supplementary Materials**

**
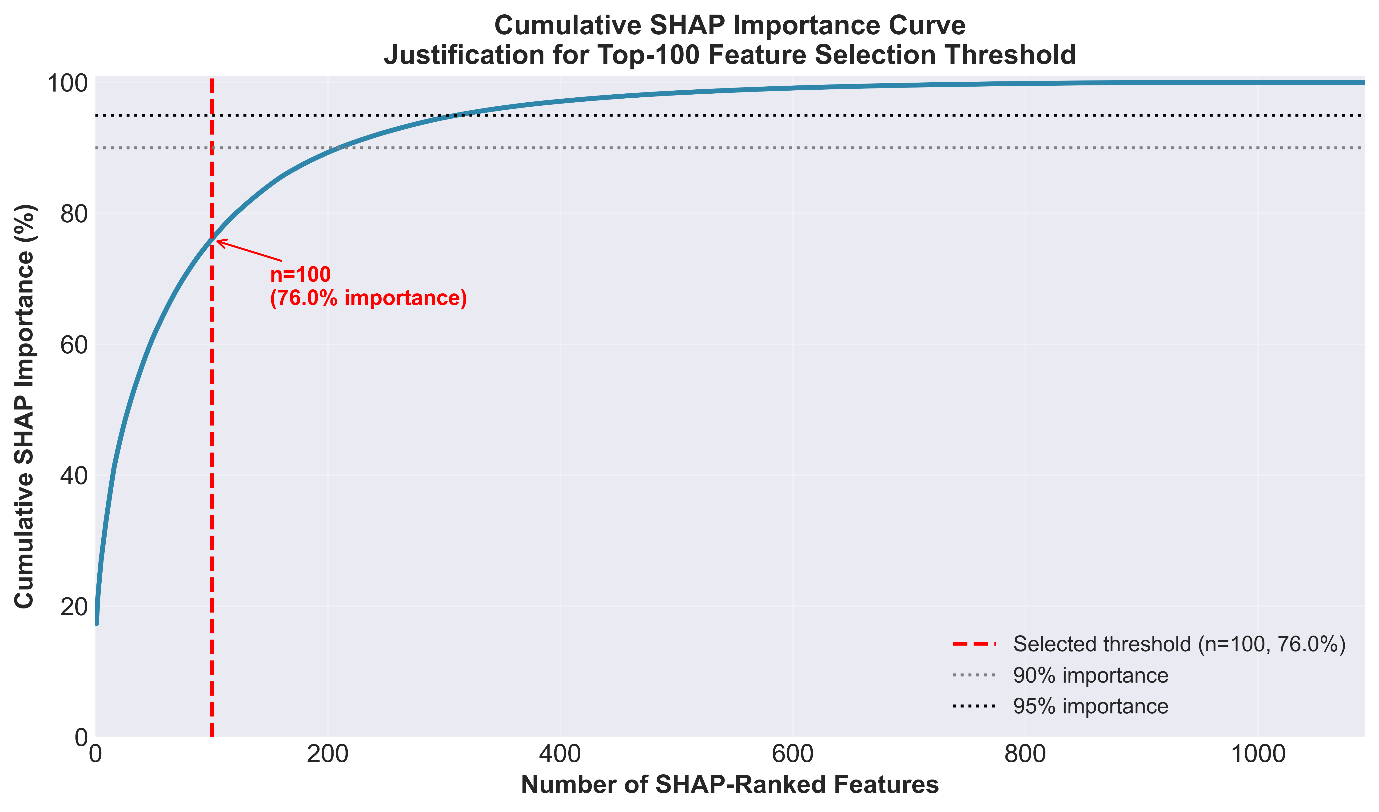
**

**Supplementary Fig S1. SHAP cumulative importance curve**

**Supplementary Table S1.** **complete masking lexicon including all masked terms, morphological variants, and abbreviations**

| **Category** | **Terms** |
| --- | --- |
| Disease Variants | wheezing, wheezed, wheezy, wheez, bronchitic, bronchial, pneumonic, bronchopneumonic, bronchial pneumonia, respiratory infection, respiratory illness, resp tract infection, upper respiratory tract infection, lower respiratory tract infection, upper respiratory infection, upper respiratory tract, chest infection, lung infection, bronchospasm, bronchoconstriction, expiratory wheeze, audible wheeze, viral wheeze, nasopharyngitis, rhinopharyngitis, common cold, pharyngitis, rhinitis, laryngitis, tonsillitis, sinusitis, otitis media, consolidation, lobar pneumonia, atypical pneumonia, bacterial pneumonia, viral pneumonia, aspiration pneumonia, community acquired pneumonia, pneumonitis, crepitations, crackles, bronchial breathing, tracheobronchitis, acute bronchitis |
| Abbreviations | urti, uri, lrt, lrti, rti, ari, pna, resp inf, resp illness, a.r.i, cap |
| Misspellings | pnemonia, pnuemonia, pnemonitis, broncitis, broncopneumonia, bronchopneumonis, broncial, wheez |
| Related Terms | infection, infective, inflamed bronchi, chest congestion, airway obstruction, respiratory distress, shortness of breath, dyspnea, tachypnea, productive cough, lung infiltrate, diagnosed with, known case of, case of, final diagnosis, provisional diagnosis, working diagnosis, impression, salbutamol, nebulization, bronchodilator, levosalbutamol, ipratropium, montelukast, budesonide |

**Table S2. Top 100 selected SHAP features with importance**

| **Sl.No** | **Feature Name** | **Importance** | **Feature Type** |
| --- | --- | --- | --- |
| 1. 1 | Age_Months | 0.12475905788157218 | Numeric |
|  | RR(cycles/min) | 0.0274999998885557 | Numeric |
|  | Breathing | 0.020667364355792348 | Numeric |
|  | combined_clinical_history__hurried | 0.011209642310585953 | Clinical Text |
|  | combined_clinical_history__chest | 0.011133062289365463 | Clinical Text |
|  | combined_clinical_history__grade | 0.010221376562078993 | Clinical Text |
|  | combined_clinical_history__indrawing | 0.008336922466193802 | Clinical Text |
|  | combined_clinical_history__cough | 0.008083059338945002 | Clinical Text |
|  | SpO2_Percent | 0.007998932586857733 | Numeric |
|  | combined_clinical_history__wet | 0.007846681407046778 | Clinical Text |
|  | combined_clinical_history__breathing | 0.007718142440943997 | Clinical Text |
|  | Weight_KG | 0.006933631600877529 | Numeric |
|  | combined_clinical_history__vomiting | 0.006898343113896737 | Clinical Text |
|  | fever_days_standardized | 0.00673737797418988 | Numeric |
|  | DEVELOPMENTAL HISTORY__normal | 0.006703477094863135 | Clinical Text |
|  | cough_cold_days_standardized | 0.006546595951717269 | Numeric |
|  | Serum Crp(mg/l)_standardized | 0.006177664292124295 | Numeric |
|  | combined_clinical_history__rigors | 0.005928300929719573 | Clinical Text |
|  | combined_clinical_history__type | 0.005869047498021385 | Clinical Text |
|  | Height_CM | 0.005322497897626065 | Numeric |
|  | Basophils_Percent | 0.005244331463566981 | Numeric |
|  | combined_clinical_history__fever | 0.004922859142543554 | Clinical Text |
|  | combined_clinical_history__child | 0.004529395016422642 | Clinical Text |
|  | Eosinophils_Percent | 0.004521882631099727 | Numeric |
|  | Ears__normal | 0.004403816090571421 | Clinical Text |
|  | POSTNATAL/NICU__nicu | 0.004181511301997609 | Clinical Text |
|  | combined_clinical_history__post | 0.004127070639201743 | Clinical Text |
|  | Serum Bicarbonate(mEq/L)_standardized | 0.003791256258994613 | Numeric |
|  | combined_clinical_history__tussive | 0.0037212116628230836 | Clinical Text |
|  | combined_clinical_history__high | 0.003445909194089414 | Clinical Text |
|  | P/A__tender | 0.0034435709522073962 | Clinical Text |
|  | combined_clinical_history__no | 0.0032475283342387074 | Clinical Text |
|  | Serum Creatinine (mg/dl)_standardized | 0.0031336951774677954 | Numeric |
|  | combined_clinical_history__not | 0.0031198644241044013 | Clinical Text |
|  | combined_clinical_history__eyes | 0.0031130841121948284 | Clinical Text |
|  | CVS__no | 0.0031037701908279433 | Clinical Text |
|  | Serum Sodium(mEq/L)_standardized | 0.0030648768344722706 | Numeric |
|  | Serum Chloride(mEq/L)_standardized | 0.0030505627171470407 | Numeric |
|  | CNS__tone | 0.002974337496841687 | Clinical Text |
|  | Neutrophils_Percent | 0.0029555861511755986 | Numeric |
|  | CNS__neurological | 0.002896047170154732 | Clinical Text |
|  | Serum Potassium(mEq/L)_standardized | 0.0028613107603570193 | Numeric |
|  | combined_clinical_history__days | 0.002826675570986392 | Clinical Text |
|  | combined_clinical_history__history | 0.0027931112593449076 | Clinical Text |
|  | CNS__no | 0.0027701743385240454 | Clinical Text |
|  | CNS__left | 0.0027448494158514728 | Clinical Text |
|  | combined_clinical_history__form | 0.0027104856741980783 | Clinical Text |
|  | combined_clinical_history__day | 0.0026909156600524306 | Clinical Text |
|  | P/A__soft | 0.002568055488583369 | Clinical Text |
|  | CNS__focal | 0.002555339318564303 | Clinical Text |
|  | Leukocyte Count Total ( /cumm) | 0.0025151647505638215 | Numeric |
|  | P/A__non | 0.002474186865334014 | Clinical Text |
|  | combined_clinical_history__apparently | 0.0023482355042705083 | Clinical Text |
|  | IMMUNIZATION HISTORY__date | 0.002266429665813643 | Clinical Text |
|  | birth_weight_kg_fixed | 0.002261268737516692 | Numeric |
|  | Lymphocytes_Percent | 0.0022300993029030804 | Numeric |
|  | combined_clinical_history__associated | 0.002200539741054237 | Clinical Text |
|  | CNS__plantar | 0.002199196374901331 | Clinical Text |
|  | CVS__murmur | 0.002141471553172866 | Clinical Text |
|  | combined_clinical_history__feeding | 0.0021241630350151237 | Clinical Text |
|  | Hemoglobin_gdL | 0.0021127368953829514 | Numeric |
|  | combined_clinical_history__uprolling | 0.0020463131788593233 | Clinical Text |
|  | IMMUNIZATION HISTORY__bcg | 0.002012721029227038 | Clinical Text |
|  | PR(bpm) | 0.001973152037308044 | Numeric |
|  | temperature_fahrenheit_standardized | 0.0019153184050442768 | Numeric |
|  | IMMUNIZATION HISTORY__immunized | 0.0019144374643850965 | Clinical Text |
|  | POSTNATAL/NICU__no | 0.0019084390972514491 | Clinical Text |
|  | CNS__nerves | 0.001859418787778409 | Clinical Text |
|  | Monocytes_Percent | 0.001822449588539968 | Numeric |
|  | CNS__right | 0.0017472391247135136 | Clinical Text |
|  | Throat__normal | 0.0017334682179997738 | Clinical Text |
|  | Platelet_Count | 0.0017070641311844894 | Numeric |
|  | POSTNATAL/NICU__cried | 0.0016973380508343453 | Clinical Text |
|  | CVS__s1 | 0.0016962625947802878 | Clinical Text |
|  | POSTNATAL/NICU__baby | 0.0016860571866352469 | Clinical Text |
|  | CNS__power | 0.0016462564005850304 | Clinical Text |
|  | DEVELOPMENTAL HISTORY__appropriate | 0.0015711163852411242 | Clinical Text |
|  | combined_clinical_history__medications | 0.0015491138351724273 | Clinical Text |
|  | DEVELOPMENTAL HISTORY__age | 0.0015126790441364092 | Clinical Text |
|  | combined_clinical_history__developed | 0.001502120063052031 | Clinical Text |
|  | CVS__s2 | 0.0014810605723113283 | Clinical Text |
|  | POSTNATAL/NICU__immediately | 0.0013946851880549248 | Clinical Text |
|  | POSTNATAL/NICU__admission | 0.0013898307917750597 | Clinical Text |
|  | CNS__signs | 0.0013223031677813079 | Clinical Text |
|  | combined_clinical_history__chills | 0.001290971888578127 | Clinical Text |
|  | combined_clinical_history__stools | 0.001266946295963865 | Clinical Text |
|  | P/A__liver | 0.0012449445874981658 | Clinical Text |
|  | combined_clinical_history__variation | 0.001229360985806023 | Clinical Text |
|  | CNS__upper | 0.0012079204603694465 | Clinical Text |
|  | combined_clinical_history__noisy | 0.001174022930148535 | Clinical Text |
|  | combined_clinical_history__loose | 0.0011290012913023695 | Clinical Text |
|  | combined_clinical_history__coryza | 0.0010847921944939394 | Clinical Text |
|  | Ears__norma1 | 0.001040692505215439 | Clinical Text |
|  | combined_clinical_history__intermittent | 0.000997762376865952 | Clinical Text |
|  | Throat__congested | 0.0008553893441534687 | Clinical Text |
|  | combined_clinical_history__relieved | 0.0008258920127951514 | Clinical Text |
|  | combined_clinical_history__difficulty | 0.0008107337238177532 | Clinical Text |
|  | combined_clinical_history__cyanosis | 0.000692253186118245 | Clinical Text |
|  | combined_clinical_history__drowsiness | 0.0005831679069095079 | Clinical Text |
|  | CNS__triceps | 0.00034047381026789324 | Clinical Text |

**Supplementary Methods**

**Software and Library Versions**

| **Library** | **Version** |
| --- | --- |
| Python | 3.13.0 |
| scikit-learn | 1.5.2 |
| XGBoost | 3.0.5 |
| SHAP | 0.48.0 |
| imbalanced-learn | 0.14.0 |
| pandas | 2.2.3 |
| numpy | 2.1.3 |
| matplotlib | 3.9.2 |

All experiments were conducted using Python 3.13.0. Compatibility with earlier Python versions was not tested.

**Model Hyperparameters**

| **Model** | **Key Hyperparameters** |
| --- | --- |
| Logistic Regression | max_iter=1000, random_state=42, solver=lbfgs |
| Random Forest | n_estimators=200, random_state=42, n_jobs=-1 |
| XGBoost | eval_metric=mlogloss, random_state=42, n_jobs=-1 |
| Stacking Ensemble | cv=5, passthrough=False, final estimator=Logistic Regression |
| SMOTE | k_neighbors=5 (default), random_state=42 |

**Cross-Validation Settings**

| **Setting** | **Value** |
| --- | --- |
| Strategy | Stratified K-Fold |
| Number of folds | 5 |
| Shuffle | True |
| Random state | 42 |
| Train-test split | 80-20 stratified |

**TF-IDF Settings**

| **Setting** | **Value** |
| --- | --- |
| Max features per column | 200 |
| Minimum document frequency | 2 |
| Lowercase | True |
| Stopwords | Custom clinical stopwords + DIAGNOSIS_TERM + UNKNOWN |

**Reproducibility Statement**

A global random seed of 42 was set using numpy.random.seed(42) prior to all experiments. All reported results are reproducible using the library versions specified above. Code is available upon request.
